# Accommodation Response Variations in University Students under High Demand for Near-Vision Activity

**DOI:** 10.3390/life12111837

**Published:** 2022-11-09

**Authors:** Concepción De-Hita-Cantalejo, María-de-los-Ángeles Benítez-Rodríguez, María Carmen Sánchez-González, María-José Bautista-Llamas, José-María Sánchez-González

**Affiliations:** Department of Physics of Condensed Matter, Optics Area, Vision Science Research Group (CIVIUS), University of Seville, 41004 Seville, Spain

**Keywords:** accommodation response, university population, near-vision activities, accommodation disorders

## Abstract

The objective of this study was to investigate accommodation changes and visual discomfort in a university student population after a period of high demand for near-vision activity. A total of 50 university students aged between 20 and 22 years were recruited. The tests performed involved positive relative accommodation (PRA), negative relative accommodation (NRA), accommodation amplitude (AA), and monocular and binocular accommodative facility (MAF and BAF). Visual discomfort was measured on a scale involving a visual discomfort questionnaire (VDQ). All accommodative variables underwent changes during the exam period; specifically, regarding NRA and PRA, 30.4% and 15.1% of the studied population, respectively, appeared to be below average. Moreover, 42.3% of the population exhibited values below average in the second measure of AA. On the other hand, a small percentage of the population was below average in MAF and BAF measurements: 3% in the monocular right eye test, 6% in the left eye test, and 9.1% in the binocular facility test. Finally, the VDQ score did not reveal a statistically significant difference between the two measurements. Prolonged near-distance work, such as a university exams period, changed all accommodation systems (amplitude of accommodation, relative accommodation, and accommodation facility). These changes influence an accommodation excess that results in blurred vision, headache, and problems with focusing.

## 1. Introduction

The human eye has the ability to adapt to different distances via changes in its refractive power, thus maintaining a clear image focused on the retina [1]. Nevertheless, accommodative response is flawed as a result of prolonged effort in near vision [2,3], resulting in accommodative and nonstrabismic binocular dysfunctions that affect the patient’s visual performance, especially during near-vision activities [4,5]. Due to excessive effort, the visual system loses efficacy and results in symptoms such as headache, blurred vision, or difficulty in focusing [6,7]. Different criteria have been used to diagnose these visual disturbances, either by observing the presence or absence of symptoms, using a symptomatology questionnaire, or carrying out an assessment test of the accommodative system [4,8]. Accommodative dysfunctions are among the most frequent causes of asthenopia symptomatology [9,10]; therefore, evaluation of the accommodative system is of great importance for the prevention of such issues. Assessment tools that provide sufficient information on the accommodative function include monocular accommodative amplitude (AA) [11,12] and monocular accommodative facility (AF) in both the phase with negative lenses and the phase with positive lenses [13]. Indirectly, by assessing binocular AA, negative and positive relative accommodation (NRA and PRA) and binocular AF are shown in both phases [8]. 

Negative relative accommodation (NRA) and positive relative accommodation (PRA) measure the maximum capacity to stimulate accommodation while maintaining unique binocular vision and indirectly provide information on fusional vergence [14,15]; therefore, determining these values aids in the diagnosis of accommodative and vergence-related problems. A low PRA value indicates accommodative insufficiency, and a low NRA value reveals accommodative excess [13,16]. The amplitude of accommodation (AA) is the maximum focusing power of the eye, measured in diopters [17]. There are different methods to perform this measurement; however, we needed to determine the most reproducible and reliable method that could detect statistically significant changes in the measured values [18]. Antona et al. conducted a study comparing different measuring methods of AA, concluding that the most repeatable method was that of negative lenses [19].

Monocular accommodative facility (MAF) measures the ability of the eye to vary its accommodative state by changing focus for a certain time, accurately and repeatedly [20]. This test is performed as a measure of visual fatigue that can be caused by accommodative dysfunctions [13]. Values outside the norm are indicative of accommodative alterations, and some authors have linked these alterations with decreased academic performance [4,21]. Therefore, most studies have focused more on children rather than adults. In addition, there are no unified criteria on clinical signs to diagnose these alterations [22]. Although some studies have obtained data on visual symptomatology in university students [23], there are a lack of studies analyzing the negative effects of excessive use of near vision. Due to the high visual demand for several hours [24], it has been observed that near-point measurements after near work are predictive of symptoms in students, which shows that a responsible mechanism produces this symptomatology after a continuous effort in near vision [25].

Visual discomfort was measured on a scale using a visual discomfort questionnaire (VDQ). This scale was considered valid and reliable for assessing the symptomatology [26].

Consequently, the purpose of this study was to investigate accommodative changes in a population of university students after an exam period, when there is a high demand for near-vision activities.

## 2. Materials and Methods

### 2.1. Design

A total of 50 patients were recruited for the study. All participants were students aged between 20 and 22 years who were given a two-stage accommodative test, the first part just after a holiday period, in September 2019, and the second part after an exam period in the first quarter, in January 2020. All tests were performed on the maximum positive refraction to result in the best visual acuity (VA) of each patient.

### 2.2. Ethical Aspects

All patients included in this work were adequately informed verbally and in writing of the tests to be performed on them. All patients signed an informed consent form prior to the start of the study. This study was conducted in accordance with the Declaration of Helsinki, and the Institutional Review Board of the University Hospital Virgen Macarena of the University of Seville approved the research.

### 2.3. Subjects

The 50 patients voluntarily attended the University of Seville. The inclusion criteria were as follows: (1) university students between the ages of 20 and 22 years, and (2) VA with or without correction of 0.0 logMAR or higher. The exclusion criteria were: (1) subjects with binocular dysfunctions, strabismus, nystagmus, or amblyopia; (2) persons with intellectual disabilities; (3) subjects who did not sign the informed consent form; (4) subjects who did not complete the survey; and (5) subjects who had undergone some type of ocular surgery.

Of the initial enrolled population, 13 students were excluded for having binocular problems. Of the 37 remaining candidates who enrolled in the study, 4 were excluded for not completing the tests. Therefore, a total of 33 students’ data were analyzed.

### 2.4. Materials and Measurements

The measurements were carried out in the laboratory of the Faculty of Pharmacy of the University of Seville. The variables with which the accommodative function was measured were: accommodative amplitude (diopters, D); negative and positive relative accommodation (D), evaluated with the ESSILOR MPH100E S/N 000104 phoropter (Essilor, Paris, France); and accommodative facility (cycles per minute), quantified with ±2 flipper lenses (Optometric Promotion, Burgos, Spain).

Before starting each test, the procedure to be performed was explained to the patient. Three measurements were made. The mean value of the three values obtained was calculated. As far as testing and regulatory values are concerned, we relied on the manual by Scheiman and Wick [8].

For the measurement of PRA, the ability to stimulate accommodation was evaluated by adding negative lenses; in the case of NRA, the ability to relax accommodation was evaluated using positive lenses. For the measurements, we used a phoropter with a correction in far vision for each patient. The optotype was located about 40 cm from the eye, and IPD was near distance. The subject was instructed to look at a line lower than that of their VA on the view card. Negative lenses were introduced for the measurement of PRA and positive lenses for the measurement of NRA, binocularly in steps of 0.25 D until the first point of blurriness occurred. The normal values set for NRA were +2 × 0.50 D and for PRA, −2.37 × 0.50 D [8].

The AA test was performed using the negative lens method. The optotype was placed in the phoropter at 40 cm with a near IPD. Negative lenses were inserted binocularly in steps of −0.25 D until the patient noticed a sustained blur, i.e., a continuous blurring. The value of AA corresponded to the power of the last lens with which the subject was able to clarify the optotype, adding the necessary accommodations to see at a 40 cm distance, i.e., 2.50 D. For the normal values, Hofstetter created three equations of AA that present its variation with the progression of age [27]. These three involve the minimum, the mean, and the maximum. In this study, the equation with the mean was used, which corresponds to the formula AA media −18.5 − 0.3 × age (years) [8]. All optometry instruments were from Optometric Promotion (Burgos, Spain)

With the MAF test, we evaluated the ability of the accommodative system to make rapid and abrupt accommodative changes, thus checking the fatigue resistance in each time and distance. During the measurements, we used a manual near card at a 40 cm distance from the patient. For both monocular (MAF) and binocular (BAF) tests, the patient was asked to look at a lower line than that of their maximum VA in the near-up optotype, which must always remain clear. The flipper was introduced with the positive lens, and we expected the participants to declare sharp vision before we turned the flipper to the negative lens, as well as the same observation from the subjects. The same process was followed for the other eye and, finally, binocularly. At the same time, we recorded the cycles per minute (cpm). The normal values set were 11 ± 5 cpm for MAF and 8 ± 5 cpm for BAF [8].

In addition, the variables that were measured to rule out binocular dysfunctions were: the magnitude of the horizontal heterophoria (Prism Diopters, Δ) [28] and the amplitude of both the positive (convergence) and negative fusional vergences (divergence) [29]. According to the value of these variables and Sheard’s criterion [30], subjects with binocular dysfunctions were excluded. Sheard’s criterion was defined as follows: in order to have a comfortable binocular vision, the value of the fusional vergence must be twice the value of the phoria.

To evaluate visual discomfort, we used the scale of visual discomfort questionnaire (VDQ), with questions regarding study time, study breaks, headache, eye pain, focus issues, blurry vision, and double vision. The VDQ [31] consisted of 23 items with a four-point scale: 0 = event never occurs; 1 = occasionally, a couple of times a year; 2 = often, every few weeks; and 3 = almost always. The items proposed by Borsting et al. [31] were well within the reading level of college students. The items of the VDQ are presented in Table 1.

### 2.5. Data Analysis

Statistical analysis was carried out with SPSS statistics 25.0 (IBM Corporation, Armonk, NY, USA). All visual acuity data were converted into Snellen formats. The Student’s *t*-test was applied for parametric-dependent variables. Effect size calculation was assessed within D of the Cohen test [32]. All statistical tests were performed with a 95% confidence interval (*p* < 0.05).

## 3. Results

### 3.1. Relative Accommodation

**Figure 1:**
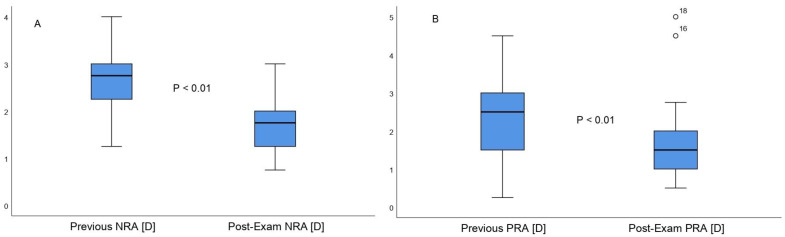
Negative Relative Accommodation (**A**) and Positive Relative Accommodation (**B**) comparative boxplots between the first and second measurement, respectively.

The pre-exams score for NRA was +2.59 ± 0.58 (+1.25 to +4.00) diopters, whereas the post-exams NRA score was +1.69 ± 0.51 (+0.75 to +3.00) diopters. Therefore, NRA was characterized by statistically significant differences between the two measurements (mean difference, 0.89 ± 0.59 diopters; 95% confidence interval, 0.68 to 1.10; t = 8.64; *p* = 0.007) (Figure 1A). Cohen´s D size was 1.64, which is considered a large effect size. The normative values for NRA, as established by Scheiman and Wick [8], were +2.00 ± 0.50 D. Our results indicated that before the exam period, 3% of the studied population were under the norm, 42.5% were within the norm, and 54.5% ere above the norm. After the exam period, 30.4% were under the norm, 63.6% were within the norm, and only 6% were above the normative values. The PRA score obtained before the exam period was −1.66 ± 1.00 (−0.50 to −5.00) diopters and reached −2.36 ± 1.01 (−0.25 to −4.50) diopters after the exam period. Therefore, PRA exhibited statistically significant differences between the two measurements (mean difference, 0.69 ± 1.10 diopters; 95% confidence interval, 0.30 to 1.08; t = 3.62, *p* = 0.009) (Figure 1B). Cohen´s D size was 0.69, which is considered a medium effect size. The normative values for PRA, as established by Scheiman and Wick [8], were −2.37 ± 1.00 D. Our results revealed that before the exam period, 48.5% of the studied population were under the norm, 45.5% were within the norm, and 6% were above the norm. Post-exams, 15.1% were under the norm, 72.8% were within the norm, and only 12.1% were above the normative values.

### 3.2. Amplitude of Accommodation

**Figure 2:**
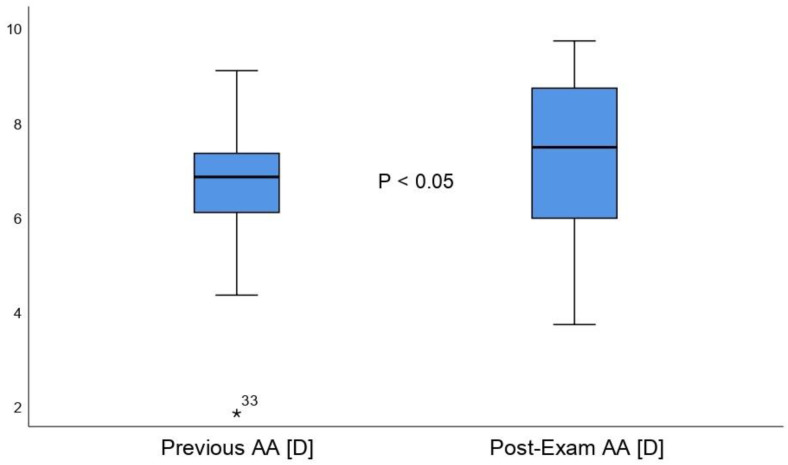
Accommodation Amplitude comparative boxplots between the first and second measurement.

The pre-exams score for AA was 6.70 ± 1.34 (2.00 to 9.25) diopters and reached 7.24 ± 1.53 (3.75 to 9.75) diopters after the exam period. Therefore, AA exhibited statistically significant differences between the two measurements (mean difference, 0.53 ± 1.38 diopters; 95% confidence interval, 0.05 to 1.02; t = 2.23, *p* = 0.03) (Figure 2). Cohen´s D size was 0.37, which is considered a medium effect size. The normative values for AA, as established by Scheiman and Wick [8], were 16 − ⅓ age ± 2.00 D (mean age was used, 8.99 ± 2.00 D). Our results demonstrated that before the exam period, 60.5% of the studied population were under the norm, and 39.5% were within the norm. Post-exams, 42.3% were under the norm, and 57.7% were within the norm. We did not observe AA scores above the normative values either pre- or post-exams.

### 3.3. Accommodative Facility

**Figure 3:**
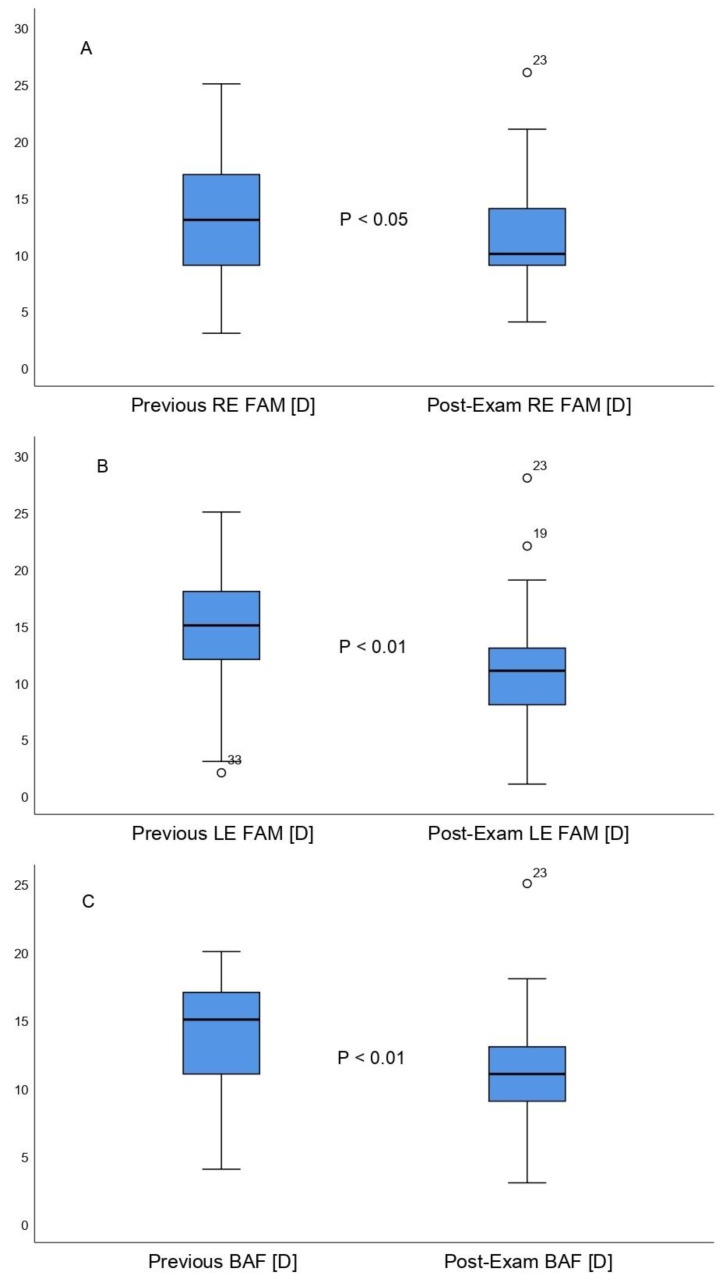
Right Eye Monocular Accommodative Facility (**A**), Left Eye Monocular Accommodative Facility (**B**), and Binocular Accommodative Facility (**C**) comparative boxplots between the first and second measurement, respectively.

The right eye MAF score obtained before the exam period was 13.24 ± 5.36 (3.00 to 25.00) cpm and reached 11.30 ± 4.44 (4.00 to 26.00) cpm after the exam period. Therefore, the right eye MAF reported statistically significant differences between the two measurements (mean difference, 1.93 ± 4.83 cpm; 95% confidence interval, 0.22 to 3.65; t = 2.23, *p =* 0.02) (Figure 3A), with a Cohen´s D size of 0.39, which is considered a medium effect size. The right eye MAF normative values, as established by Scheiman and Wick [8], were 11 ± 5 cpm. Our results reported that before the exam period, 6.1% of the studied population were under the norm, 66.8% were within the norm, and 27.2% were above the norm. After the exam period, 3% were under the norm, 88% were within the norm, and 9% were above the normative values. The left eye MAF score obtained before the exam period was 14.27 ± 4.95 (2.00 to 25.00) cpm and decreased to 11.42 ± 5.20 (1.00 to 28.00) cpm post-exams. Therefore, the left eye MAF differences between the two measurements were statistically significant (mean difference, 2.84 ± 4.61 cpm; 95% confidence interval, 1.21 to 4.48; t = 3.54, *p* = 0.009) (Figure 3B), with a Cohen´s D size of 0.56, which is considered a medium effect size. The left eye MAF normative values, as established by Scheiman and Wick [8], were 11 ± 5 cpm. Our results revealed that before the exam period, 6% of the studied population were under the norm, 63.7% were within the norm, and 30.3% were above the norm. Post-exams, 6% were under the norm, 82% were within the norm, and 12% were above the normative values. Additionally, the pre-exams BAF score was 13.51 ± 4.36 (4.00 to 20.00) cpm and decreased to 10.96 ± 4.53 (3.00 to 25.00) cpm after the exam period. Therefore, BAF was also characterized by statistically significant differences between the two measurements (mean difference, 2.54 ± 4.72 cpm; 95% confidence interval, 0.82 to 4.22; t = 3.09, *p* = 0.004) (Figure 3C), with a Cohen´s D size of 0.57, which is regarded as a medium effect size. The BAF normative values, as established by Scheiman and Wick [8], were 10 ± 5 cpm. Our results demonstrated that before the exam period, 3% of the studied population were under the norm, 57.6% were within the norm, and 39.4% were above the norm. Post-exams, 9.1% were under the norm, 78.9% were within the norm, and 12% were above the normative values.

### 3.4. Visual Discomfort

Finally, the visual discomfort questionnaire (VDQ) score obtained before the exam period was 17.84 ± 1.08 (4.00 to 31.00) and 17.93 ± 0.96 (6.00 to 28.00) post-exams. Therefore, the VDQ score did not present statistically significant differences between the two measurements (mean difference, −0.09 ± 3.80 points; 95% confidence interval, −1.43 to 1.25; t = −0.13, *p* = 0.892), with a Cohen´s D size of 0.08, which is regarded as a small effect size. No further statistical analysis related to VDQ score was conducted.

## 4. Discussion

The results of our study on young university students indicated that all the accommodative variables measured underwent changes after the exam period. Measures of relative accommodation, both positive and negative, accommodative facility, and amplitude of accommodation were carried out. These tests have been used by several authors to identify the type of disorder [33,34]. This system developed by Scheiman and Wick is commonly used as a reference for the classification, diagnosis, and treatment of accommodative disorders [8].

The mean NRA was (+2.59 ± 0.58) D pre-exams and (+1.69 ± 0.51) D post-exams. In addition, the measure PRA was (−1.66 ± 1.00) D pre-exams and (−2.36 ± 1.01) D post-exams in our study, respectively. Negative relative accommodation (NRA) and positive relative accommodation (PRA) are used as diagnostic tests of accommodative disorders. Many authors establish a relationship between low NRA and PRA values and accommodative excess and insufficiency, respectively [24]. García et al. [35] determined that high PRA values do relate to disorders associated with accommodative excess, and they considered this alteration as a diagnostic measure of this anomaly. A low value of PRA indicates that the patient does not admit the introduction of negative lenses. The subject is not able to increase his/her accommodation to a situation that may be related to an accommodative insufficiency. A low value NRA indicates that the subject does not admit the binocular interposition of positive lenses, because he is not able to relax the accommodation; it probably indicates accommodative excess.

Other studies [11,36] on university students in which measures of accommodative parameters were taken have observed significant changes in PRA, which were associated with accommodative dysfunctions. The same observation was made [37] in young workers who perform daily near-vision work (high PRA values after increased near work). Thus, the variation in PRA values coincides with the data in our study collected in the second measurement. Although our values are lower, they could represent the beginning of an accommodative excess in the study population.

Concerning AA, the results of the present work seem to be in the same line as those obtained by different authors who concluded that AA increases after many hours of work in near vision [22,38]. The mean AA was (6.70 ± 1.34) pre-exams and (7.24 ± 1.53) D post-exams. Considering different studies, we decided to use the low accommodative amplitude and high accommodation amplitude as the clinical sign for diagnosing accommodative insufficiency and excess [11]. Literature-reported prevalence of accommodative disorders varies greatly due to the lack of standardization in the type of subjects enrolled and clinical diagnostic tests employed. However, our results are in agreement with other studies that state that increased near-visual activity increases AA and produces an accommodative excess [39].

Further, to determine the state of the accommodative function, it is necessary to assess the monocular accommodative facility (AF) both in the phase with positive lenses and in the phase with negative lenses, as well as binocular AF in both phases. The fundamental clinical sign for accommodative excess was failing monocular accommodative facility with positive lenses.

MAF values were also modified, although to a lesser extent. MAF values were very similar before and after the exam period, with 27.2% of the right eye (OD) and 30.3% of the left eye (OI) above the norm before the exam period, 3% of the OD and 6% of the OI below the norm, and 88% of the OD and 82% of the OI within the average after the exam period. BAF results were 57.6% within the norm and 39.4% above the norm before the exam period, and 78.9% within the norm, 12% above the norm, and 9.1% below the norm after the exams. Porcar et al. [39] analyzed the accommodative and binocular dysfunctions caused by the use of computers. They included 89 patients who underwent vergence and accommodative tests; all had been using digital screens an average of 5 ± 9 h/day, and none was diagnosed with visual system disturbance. Regarding MAF, 24% of the patients failed focusing positive lenses, 8% failed focusing negative lenses, and 3% with both. Based on all the data obtained, they concluded that the patients analyzed were more prone to accommodative excess, especially considering that, currently, students use computers for longer durations. This observation confirms the result of our study.

Finally, the VDQ obtained 17.84 ± 1.08 (4.00 to 31.00) points before the exam period and 17.93 ± 0.96 (6.00 to 28.00) post-exams. Therefore, the VDQ did not report statistically significant differences between the two measurements (t = −0.13, *p* = 0.892). Borsting et al. [23] conducted a study in which they assessed symptoms of visual discomfort in 23 university students for one year using the same scale, reporting no statistically significant differences between the first and second measure of their survey. They concluded that the symptomatology was stable in most patients, supporting the data of our study. On the one hand, the significantly subjective measurements changes were connected with blurred vision, headache, and accommodation problems. All accommodation variables became worse after the period exam. However, on the other hand, the objective questionnaire was a self-patient sensation report. The patients do not perceive these accommodation alterations when filling in the questionnaire; therefore, it supposes an element that increases severity. This indicates that they do not notice symptoms, and it is necessary to carry out periodic visual check-ups of the students during exam periods.

This study presents some limitations that should be considered. One of these is the small sample size, as several patients were left out due to the exclusion criteria. Therefore, studies with a larger sample size would be needed. On the other hand, at the second stage of the study, some of the recruited patients did not show up for the second appointment, thus further decreasing the total number of measures. Another limitation is that the studied population included students of a certain age range; consequently, this study should be performed in both adults over 25 years of age and in children.

## 5. Conclusions

Prolonged near-distance work, such as a university exams period, changes all accommodation systems (amplitude of accommodation, relative accommodation, and accommodation facility). These changes could influence an accommodation excess that results in blurred vision, headache, and problems with focusing.

## Figures and Tables

**Table 1:** Item list of the Visual Discomfort Questionnaire by Borsting et al. [31].

Question	Item
When reading, do the words or letters in the words ever appear to spread apart?	movement/fading
Do you ever have difficulty reading the words on a page because they begin to flicker or shimmer?	movement/fading
Does the white background behind the text ever appear to move, flicker, or shimmer, making the letters hard to read?	movement/fading
Do you ever have difficulty seeing more than one or two words on a line in focus?	blur/diplopia
When reading, do the words on the page ever begin to move or float?	movement/fading
When you are reading a page that consists of black print on white background, does the background ever appear to overtake the letters, making them hard to read?	movement/fading
When reading, do the words on a page of clear text ever appear to fade into the background, then reappear?	movement/fading
Do the letters on a page ever appear as a double image when you are reading?	blur/diplopia
When reading black print on a white background, do you ever have to move the page around or continually blink to avoid glare that seems to come from the background?	movement/fading
Do you ever get a headache from reading a newspaper or magazine with clear print?	headache
Do you have to move your eyes around the page or continually blink or rub your eyes to keep the text easy to see when you are reading?	blur/diplopia
When reading, do you ever have to squint to keep the words on a page of clear text from going blurry or out of focus?	blur/diplopia
Do the letters on a page of clear text ever go blurry when you are reading?	blur/diplopia
When reading, do you ever have difficulty keeping the words on the page of clear text in focus?	blur/diplopia
Do your eyes ever feel watery, red, sore, strained, tired, dry, or gritty after you have been reading a newspaper or magazine with clear print?	sore
Do your eyes ever feel watery, red, sore, strained, tired, dry, or gritty, or do you rub them a lot, when viewing a striped pattern?	sore
Do you have to use a pencil or your finger to keep from losing your place when reading a page of text in a novel or magazine?	rereading
As a result of any of the above difficulties, do you find reading a slow task?	rereading
How often do you get a headache when working under fluorescent lights?	headache
Do your eyes ever feel watery, red, sore, strained, tired, dry, or gritty, when working under fluorescent lights?	sore
When reading under fluorescent lights or in bright sunlight, does the glare from the bright white glossy pages cause you to continually move the page around so that you can see the words clearly?	glare
When reading, do you ever unintentionally re-read the same words in a line of text?	rereading
When reading, do you ever unintentionally re-read the same line?	rereading

## Data Availability

The data presented in this study are available on request from the corresponding author. The data are not publicly available due to their containing information that could compromise the privacy of research participants.
